# Prevalence and correlates of being bullied among in-school adolescents in Beijing: results from the 2003 Beijing Global School-Based Health Survey

**DOI:** 10.1186/1744-859X-7-6

**Published:** 2008-04-02

**Authors:** Alice Hazemba, Seter Siziya, Adamson S Muula, Emmanuel Rudatsikira

**Affiliations:** 1 Department of Community Medicine, University of Zambia Medical School, Lusaka, Zambia; 2 Department of Community Health, University of Malawi, Blantyre, Malawi; 3 Departments of Global Health, Biostatistics and Epidemiology, School of Public Health, Loma Linda University, Loma Linda, CA, USA

## Abstract

**Background:**

Bullying has public health importance. It has been reported that both the victims and perpetrators of bullying are more likely to have suicidal ideation and other suicidal behaviours. Moreover, bullying can be a precursor for school violence and can contribute to poor academic performance. The purpose of the study was to raise awareness on the subject in China. We, therefore conducted an analysis of secondary data to determine the prevalence and correlates of having been bullied among in-school adolescents.

**Methods:**

The data was taken from the Beijing Global School-Based Health Survey conducted in 2003. A weighted analysis to reduce bias due to differing patterns of non-response was conducted using statistical software (SPSS version 14.0). We conducted a backward logistic regression analysis to determine independent predictors for being bullied.

**Results:**

Out of a total of 2,348 in-school adolescents who participated in the survey, 20% (23% males, and 17% females) reported having been bullied. Risk factors for having been bullied were loneliness (adjusted odds ratio (AOR) = 1.44; 95% confidence interval (CI) 1.42–1.45), being worried (AOR = 1.30; 95% CI 1.29–1.32), being sad or having feelings of hopelessness (AOR = 1.21; 95% CI 1.19–1.22), smoking cigarettes (AOR = 1.09; 95% CI 1.08–1.11), drinking alcohol (AOR = 1.31; 95% CI 1.29–1.32), and being truant (AOR = 1.24; 95% CI 1.22–1.27). Meanwhile protective factors were having close friends (AOR = 0.84; 95% CI 0.83–0.86), receiving parental supervision (AOR = 0.80; 95% CI 0.80–0.81), and ever been drunk (AOR = 0.86; 95% CI 0.84–0.87).

**Conclusion:**

We believe the results of this study will raise awareness among school health practitioners and administrators, paediatric psychiatrists and psychologists on the prevalence and correlates of bullying among adolescents in Beijing, China.

## Background

Bullying is a major source of victimisation among youth [1-6]. Although there has been growing interest in the topic in the last 20 years [1-8], studies that have been reported have mainly come from Europe and the United States. There are also some reports from Hong Kong, India and Korea [9-12].

Wong has reported that if left uncurbed, bullying can be a precursor of school violence and delinquency [13]. Kim *et al*. [9] reported that both the victims and perpetrators of bullying were more likely to report suicidal ideation and other suicidal behaviours than those not associated with bullying. In a US study reported by Nansel *et al*. [14] in which 15,686 students in grades 6 to 10 were studied, bullying others and being bullied were consistently associated with self-report of weapon carrying, weapon carrying in school, physical fighting, and being injured in a physical fight. Harel previously reported that bullying of girls in Kenya was contributing to low academic achievement [15]. Therefore, bullying has important public health significance. While the majority of reports on bullying are from studies conducted among youth and in-school children, it is also important to recognise that bullying may also occur among adults, and also in out-of-school settings. Bullying may occur at work [16,17] or within prison settings [18,19]. For the current study however, our interest is in adolescents in in-school settings.

Despite the fact that there is growing interest on adolescents' in-school bullying, there are limited data from the emerging economy nations such as China. In order to raise awareness on the subject in China, we therefore conducted a secondary analysis of the Beijing Global School-Based Health Survey (2003) to investigate the prevalence and correlates of having been bullied within the last 30 days among in-school adolescents.

## Methods

### Study design

This study involved secondary analysis of existing data available from the Beijing Global School-Based Health Survey (GSHS) conducted in 2003. The GSHS was developed by the World Health Organization (WHO) in collaboration with The United Nations Children's Fund (UNICEF), The United Nations Educational, Scientific and Cultural Organization (UNESCO) and The United Nations Joint Programme on HIV/AIDS (UNAIDS), with technical assistance from the Centers for Diseases Control and Prevention (CDC), Atlanta, GA, USA. The GSHS aims to provide data on health and social behaviours among in-school adolescents. In Beijing, students from grades 1 to 4 of junior middle school were recruited.

### Sampling

The 2003 Beijing GSHS used a two-stage probability sampling technique. In the first stage of sampling, the sampling frame consisted of all junior middle schools. Schools were selected with probability proportional to school enrolment size. In the second step, a random sample of classes in the selected schools was obtained. All students in the selected classes were eligible to participate in the survey. All the 25 selected schools participated in the survey. Altogether, 2,348 students were enrolled into the survey with a response rate of 99%.

### Ethical issues

The privacy of students was protected by allowing for anonymous and voluntary participation [20]. The questionnaire was self-completed by students within one class period.

### Data analysis

Data analysis was performed using SPSS version 14.0 software. A weighting factor was used in the analysis to reflect the likelihood of sampling each student and to reduce bias by compensating for differing patterns of non response. The weight used for estimation is given by the following formula:

W = W1 * W2 * f1 * f2 *f3 *f4

Where W1 = the inverse of the probability of selecting the school, W2 = the inverse of the probability of selecting the classroom within the school, fl = a school-level non response adjustment factor calculated by school size category (small, medium, large), f2 = a class-level non response adjustment factor calculated for each school, f3 = a student-level non response adjustment factor calculated by class, and f4 = a post stratification adjustment factor calculated by grade.

We used the following questions for the outcome of interest and some of the explanatory variables: "During the past 30 days, on how many days were you bullied?", with the responses 0 days, 1 or 2 days, 3 to 5 days, 6 to 9 days, 10 to 19 days, 20 to 29 days, all 30 days. As we were interested in any history of having been bullied, we recoded the variable to a binary variable with responses of zero for 0 days and one for any number of days bullied. A follow up question indicated how "being bullied" was defined in the survey: "During the past 30 days, how were you bullied most often?". The possible responses were: "I was not bullied during the past 30 days", "I was hit, kicked, pushed, shoved around, or locked indoors", "I was made fun of because of my race or colour", "I was made fun of because of my religion", "I was made fun of with sexual jokes, comments, or gestures", "I was left out of activities on purpose or completely ignored", "I was made fun of because of how my body or face looks", and "I was bullied in some other way". The other questions that we used in the analysis were: "During the past 12 months, how often have you felt lonely?", "During the past 12 months, how often have you been so worried about something that you could not sleep at night?", "During the past 12 months, did you ever feel so sad or hopeless almost every day for two weeks or more in a row that you stopped doing your usual activities?", "How many close friends do you have?", "During your life, how many times did you drink so much alcohol that you were really drunk?". We also used data on age and gender (sex). Tian *et al*. [20] reported that the data arising from the above variables are of acceptable quality.

We obtained frequencies as estimates of prevalence rates. We conducted a backward logistic regression analysis to estimate the associations between relevant predictor variables and the outcome. The predictor variables were identified from the literature as possible factors that may be associated with having been a victim of bullying [11,21-27]. We report unadjusted odds ratios for selected predictor variables while considering having been bullied in the past month as a dependent variable. We also report results from a multivariate analysis (adjusted odds ratios) to determine independent predictors for the outcome.

## Results

### Description of the study sample

A total of 2,348 in-school adolescents participated in the Beijing Global School-Based Survey of 2003. For those study participants whose data were available, 32.6% were of age 14 years, 49.5% were females, and 20% reported having been bullied in the past month (23% among boys versus 17% among girls). Further description of the study sample is given in Table 1.

**Table 1:** Sample description of study participants in the Beijing 2003 Global School-Based Survey

**Factor**	Total, n (%)*	Male, n (%)*	Female, n (%)*
Age			
≤12	274 (12.6)	125 (11.8)	142 (12.8)
13	588 (25.5)	256 (23.0)	332 (28.3)
14	788 (32.6)	373 (32.1)	415 (33.4)
15	560 (23.5)	293 (25.7)	267 (21.4)
16+	136 (5.8)	84 (7.5)	52 (4.2)
Sex			
Male	1131 (50.5)	-	-
Female	1210 (49.5)	-	-
Loneliness			
Yes	1354 (57.2)	607 (53.6)	746 (61.2)
No	990 (42.8)	521 (46.4)	463 (38.8)
Worried			
Yes	1169 (49.3)	514 (45.4)	652 (53.4)
No	1174 (50.7)	613 (54.6)	557 (46.6)
Sad/hopeless			
Yes	442 (19.0)	222 (19.9)	217 (18.0)
No	1888 (81.0)	895 (80.1)	989 (82.0)
Had close friend			
Yes	2163 (92.4)	1044 (92.7)	1114 (92.2)
No	177 (7.6)	81 (7.3)	94 (7.8)
Smoked cigarettes			
Yes	205 (9.2)	177 (16.2)	27 (2.1)
No	2101 (90.8)	917 (83.8)	1178 (97.9)
Drank alcohol			
Yes	278 (12.9)	187 (18.1)	91 (7.8)
No	1917 (87.1)	854 (81.9)	1056 (92.2)
Ever been drunk			
Yes	184 (8.0)	132 (11.8)	52 (4.2)
No	2139 (92.0)	988 (88.2)	1144 (95.8)
Missed classes			
Yes	112 (4.9)	70 (6.3)	41 (3.5)
No	2227 (95.1)	1055 (93.7)	1166 (96.5)
Parental supervision			
Always	585 (25.3)	265 (23.7)	318 (26.8)
Not always	1752 (74.7)	858 (76.3)	889 (73.2)
Bullied			
Yes	439 (20.0)	244 (23.0)	194 (17.0)
No	1777 (80.0)	816 (77.0)	955 (83.0)

*n = unweighted frequency; weighted percentages were used.

### Factors associated with being a victim of bullying

Bivariate and multivariate analyses produced similar results (Table 2), except for ages <13 and 13 years, and ever been drunk. While odds ratios for <13 and 13 years indicated that these groups were protective in bivariate analysis, these ages were risk factors in multivariate analysis. Ever been drunk was a risk factor in bivariate analysis but was a protective factor in multivariate analysis. We highlight the results obtained in the multivariate analysis.

**Table 2:** Factors associated with being bullied

**Factor**	**Crude odds ratio (95% CI)**	**Adjusted odds ratio (95% CI)**
Age		
≤12	0.98 (0.96–1.00)	1.29 (1.26–1.32)
13	0.92 (0.90–0.93)	1.04 (1.02–1.05)
14	1.23 (1.21–1.24)	1.20 (1.18–1.22)
15	0.95 (0.93–0.96)	0.79 (0.77–0.80)
16+	1	1
Sex		
Male	1.21 (1.20–1.22)	1.21 (1.20–1.22)
Female	1	1
Loneliness		
Yes	1.58 (1.56–1.59)	1.44 (1.42–1.45)
No	1	1
Worried		
Yes	1.43 (1.42–1.45)	1.30 (1.29–1.32)
No	1	1
Sad/hopelessness		
Yes	1.43 (1.41–1.44)	1.21 (1.19–1.22)
No	1	1
Had close friends		
Yes	0.75 (0.74–0.76)	0.84 (0.83–0.86)
No	1	1
Smoked cigarettes		
Yes	1.28 (1.26–1.29)	1.09 (1.08–1.11)
No	1	1
Drank alcohol		
Yes	1.45 (1.43–1.46)	1.31 (1.29–1.32)
No	1	1
Ever been drunk		
Yes	1.29 (1.28–1.31)	0.86 (0.84–0.87)
No	1	1
Missed classes		
Yes	1.49 (1.47–1.52)	1.24 (1.22–1.27)
No	1	1
Parental supervision		
Yes	0.70 (0.70–0.71)	0.80 (0.80–0.81)
No	1	1

Compared to older adolescents of age 16 years or more, younger adolescents (<15 years) were more likely to be bullied. However, adolescents of age 15 years were less likely to be bullied. Male adolescents were 21% (adjusted odds ratio (AOR) = 1.21; 95% confidence interval (CI) 1.20–1.22) more likely to be bullied compared to female adolescents.

The other risk factors that were identified in the analysis were loneliness (AOR = 1.44; 95% CI 1.42–1.45), being worried (AOR = 1.30; 95% CI 1.29–1.32), and being sad or having feelings of hopelessness (AOR = 1.21; 95% CI 1.19–1.22). Adolescents who had close friends were 16% (AOR = 0.84; 95% CI 0.83–0.86) less likely to be bullied compared to adolescents who had no close friends. Adolescents who reported having received parental supervision were 20% (AOR = 0.80; 95% CI 0.80–0.81) less likely to be bullied.

Adolescents who smoked cigarettes were 9% (AOR = 1.09; 95% CI 1.08–1.11) more likely to be bullied compared to non-smokers. While adolescents who drank alcohol were 31% (AOR = 1.31; 95% CI 1.29–1.32) more likely to be bullied compared to adolescents who did not take alcohol, adolescents who ever had been drunk were 14% (AOR = 0.86; 95% CI 0.84–0.87) less likely to be bullied. Finally, adolescents who reported having been truant were 24% (AOR = 1.24; 95% CI 1.22–1.27) more likely to be bullied compared to adolescents who never missed classes.

## Discussion

Our study reports an overall prevalence of being bullied over the past 30 days to the survey among Beijing students of 20%. Males were more likely to have reported being victims of bullying than females (23% versus 17%, respectively). In South Africa, Liang *et al*. [27] reported a 12 months prevalence of being a victim of bullying of 19.3% among adolescents. Kepenekci and Çınkır [25] reported that in a sample of 692 Turkish high school students, all students reported having been bullied in the current academic year. In this Turkish study, 35.0% reported that they had been bullied verbally, 35.5% had been bullied physically, 28.3% had been bullied emotionally, and 15.6% had been bullied sexually, at least once during the academic year. That having been a victim of bullying was universal appears surprising. However, this may have resulted from how bullying was defined. Bullying comprises different forms including verbal (such as being made fun of, and teasing) and physical forms (such as hitting, kicking, pushing, and being locked indoors) [28].

Our study found that males were more likely to have been victims of bullying compared to females. Munni and Mahli [29] reported that females were more likely to be victims of bullying. In the sample of Turkish high school students, boys were more likely to have suffered physical bullying including kicking/slapping, assault with a knife, and rude physical jokes than girls. Kshirsagar *et al*. however reported that the prevalence of bullying was the same among boys and girls in co-education schools in India [11].

We found that adolescents who reported having been bullied were also more likely to have smoked, used alcohol, ever gotten drunk, felt hopeless, been worried, felt sad, and been truant. These factors have been reported to be associated with bullying victimization. However due to the cross sectional nature of the study, it is not possible to determine whether these factors are the consequences of having been bullied or are in the causal pathway. Bond *et al*. [30] reported in a prospective study that girls who were once victims of bullying were more likely to have depression. In another prospective study, Gladstone *et al*. [31] reported that victims of bullying suffered anxiety later in life.

### Limitations of the study

Our study has a number of limitations. Firstly, data were collected through self-reports. As in all such studies, both inadvertent and deliberate misreporting is a concern. However, the collection of data in this study was anonymous. This may have discouraged deliberate misreporting. This study asked participants whether they had been bullied. The information that was given depended on each adolescent's understanding of bullying. In a setting where the subject matter has not been adequately addressed in common language usage, the problem of having diverse interpretations amongst study participants multiplies. Although the study was conducted in Chinese so as to minimise misunderstanding of terms, the translation of the GSHS questionnaire may have altered the meaning or interpretation of questions or responses. This would make it difficult to compare the current findings with findings from other settings. However, Tian *et al*. [20] reported that the data used in the current study are of acceptable quality.

## Conclusion

We have for the first time reported the prevalence of bullying among in-school adolescents in Beijing, China. We believe this report will raise awareness on the problem of bullying behaviours among students in this setting. Efforts to prevent and control bullying behaviours need to consider the factors that are associated with the behaviour.

## Competing interests

The author(s) declare that they have no competing interests.

## Authors' contributions

AH conducted data analysis, participated in the interpretation of the results, and drafting of the manuscript. SS conducted a re-analysis of data, participated in the interpretation of results, and drafting of the manuscript. ASM participated in the interpretation of the results, and drafting of the manuscript. ER participated in the interpretation of the results, and drafting of the manuscript. All authors read and approved the final manuscript.
